# Trends in Income-Related Gaps in Enrollment in Early Childhood Education: 1968 to 2013

**DOI:** 10.1177/2332858416648933

**Published:** 2016-05-16

**Authors:** Katherine Magnuson, Jane Waldfogel

**Affiliations:** University of Wisconsin-Madison; Columbia University

**Keywords:** preschool, early education, income inequality

## Abstract

We use data from the 1968–2013 October Current Population Survey to document trends in 3- and 4-year-old children’s enrollment in center-based early childhood education, focusing on gaps in enrollment among children from low-, middle-, and high-income families. We find that income-related gaps in enrollment widened in the 1970s and 1980s but appear to have plateaued or narrowed for succeeding cohorts. These patterns are consistent with recent trends in income-related gaps in school achievement.

The academic achievement gap between children from the lowest- and highest-income families in the United States rose from the 1970s to the 1990s and is now larger than the Black-White achievement gap (Reardon, 2011). Although the income-related gap appears to have narrowed somewhat thereafter (Bassok & Latham, 2014; Magnuson & Duncan, in press; Reardon & Portillo, 2015), it remains substantial. Because this income-related gap in academic skills is already present when children start school (see, e.g., Bradbury, Corak, Waldfogel, & Washbrook, 2015), it is important to understand whether and how it relates to disparities in children’s experiences in their earliest years. To that end, in this article, we explore trends in income-related gaps in a key element of children’s early experiences—their enrollment in center-based early childhood education.

Motivating our analysis are three stylized facts: Early childhood education, particularly when it is high quality, is associated with higher levels of school readiness and subsequent achievement; historically, children from low-income families have been less likely than their more affluent peers to be enrolled in such programs; and the federal government and state and local governments play an important role in subsidizing early childhood education, particularly for low-income families (Magnuson & Waldfogel, 2005). If income-related gaps in enrollment have widened over time, due to lagging public investments or for other reasons (or if early childhood education has become more consequential for later achievement), then such trends may have played a role in increasing income-related gaps in school achievement. Conversely, if gaps in enrollment in early childhood education have narrowed, due to increased public investments or for other reasons (or if early childhood education has become less important for later achievement), then such trends may have acted to reduce gaps in achievement.

Using data on family income and school enrollment from the annual October Current Population Survey (CPS), we document the trends in enrollment in center-based early childhood education, which we refer to as *preschool*, for 3-and 4-year-old children from 1968 to the present, to examine to what extent gaps in enrollment among low-, middle-, and high-income children have widened, narrowed, or remained the same. We build on an earlier analysis that covered the period of 1968 to 2000 by Bainbridge and colleagues (Bainbridge, Meyers, Tanaka, & Waldfogel, 2005), extending it to 2013 and examining more fine-grained income categories. To put these results in context, we also provide some information on racial/ethnic gaps in enrollment over the same period. The October CPS data do not include information on quality but do allow us to look at whether a child was enrolled in a public or private program.

## Background

That poor children perform less well than children from more affluent families at school entrance is well understood. The specific role of income in causing these differences has been more controversial, but an increasing number of rigorous studies indicate that early childhood poverty shapes children’s academic outcomes (Magnuson & Votruba-Drzal, 2009). The role of differences in family income at the higher end of the income distribution is far less clear, and yet work by Reardon (2011) finds that the gap in achievement between the wealthy (90th income percentile) and the poor (10th income percentile) has grown over time. In seeking to understand this pattern of diverging achievement, Reardon (2011) concluded that trends of increasing income inequality do not offer a simple explanation; rather, it appears that income is itself more consequential for achievement, likely because more affluent parents invest more resources in their young children’s development.

There are many important ways in which parents invest in the development of their young children, but early childhood care and education are often the most costly and effective investments. In the years from infancy to school entry, children experience a variety of care and education arrangements—ranging from care by their parents (we refer to this as *parental care*) to care by a relative, nanny, or babysitter in the child’s own home or in a babysitter’s or family day care provider’s home (we refer to this as *informal care*) to care in a school or center-based setting, such as a day care center, nursery school, preschool, Head Start program, or prekindergarten (we refer to this interchangeably as *early education, center-based care*, or *preschool*).

Most children who are enrolled in preschool attend private programs, for which their parents pay fees. These programs may be nonprofit or for-profit and may operate on a full- or part-time basis. Some preschool programs operate full day and year-round, whereas others offer only part-time or school year programming. Low-income employed parents may receive help that offsets some of the costs through publicly funded child care subsidies; other families with employed parents may also receive financial assistance through various tax provisions, including the child and dependent care tax credit and the dependent care assistance plan. As we discuss in more detail below, a growing share of 3- and 4-year-olds attends publicly funded preschools, most commonly federally funded Head Start programs and prekindergarten programs funded by state or local school districts.

Preschool is an expensive form of nonparental care, typically costing far more than other informal care arrangements. A report by Child Care Aware of America (2014) found that in 2013, the average annual cost for center-based care for an infant was higher than a year’s tuition and fees at a 4-year public college in 31 states. Costs for center-based care vary across states and markets and with child age. In 2013, for example, the average cost of full-time center-based care for a 4-year-old ranged from $4,515 in Tennessee to just over $12,320 in Massachusetts. The costs for child care are largely driven by standard microeconomic forces, with the variation in prices being explained by the costs of labor and regulatory requirements, as well as the balance of supply and demand within a market (Blau, 2001). Given the high costs, it is not surprising that the use of center-based care increases with family income (Rigby, Ryan, & Jeanne Brooks-Gunn, 2007) and is more responsive to price and income changes than the use of other types of child care (Blau & Hagy, 1998). However, it is also important to note that the availability of subsidies and other programs for low-income families means that their use of center-based care may be as high or higher than that of middle-income families who do not have access to such supports (see, e.g., Fuller & Liang, 1996).

Despite the relatively high cost of preschool, the share of children who experience preschool at age 3 or 4 years has increased dramatically over time. Bainbridge and colleagues (2005) found that from 1968 to 2000, enrollment rates increased from 8% to 39% for 3-year-olds and from 23% to 65% for 4-year-olds. These increases in preschool enrollment were not only due to increases in maternal employment, as enrollment increased even among mothers who did not participate in the formal labor market. Although increases in preschool were evident throughout the income distribution, Bainbridge and colleagues found that income-related gaps in enrollment were persistent and sizable over this period. Between 1978 and 2000, the gap in preschool enrollment between families in the bottom quartile of the income distribution and those in the top quartile averaged between 15 and 18 percentage points among 3-year-olds and 13 and 21 percentage points among 4-year-olds. Enrollment gaps were considerably smaller when children in the second-highest quartile of family income were compared with those in the bottom quartile, and children from families with incomes in the second-lowest quartile did not have different rates of preschool participation than did those in the bottom quartile. Bainbridge and colleagues also found that income-related enrollment gaps for 4-year-olds were at their peak in the 1980s and declined somewhat during the 1990s.

Enrollment in early childhood education, of course, is not purely a private matter. Through programs such as Head Start, the federal government subsidizes enrollment in early childhood education, particularly for low-income children, and states and localities too play an active role in this area, through programs such as prekindergarten, which have grown considerably in recent decades. An analysis by Magnuson, Meyers, and Waldfogel (2007) found that the decline in income-related preschool enrollment gaps during the 1990s was, at least in part, due to the expansion of public funding to support low-income children’s participation in center-based programs, including Head Start.

Public funding has expanded further since 2000, suggesting that we may expect to see further narrowing of gaps in enrollment in the year after 2000. Expanded federal funding for Head Start has meant that, after holding constant at roughly 400,000 to 450,000 children in the 1980s, Head Start enrollment increased from just under 550,000 in 1990 to >850,000 in 2000 and >925,000 in 2014 (Office of Head Start, 2014). Enrollment in state-funded prekindergarten has increased even more dramatically in the past decade, doubling from 14% of 4-year-olds in 2002 to 29% in 2014 (National Institute for Early Education Research, 2004).

The spread of universal prekindergarten has raised the possibility that it may be “crowding out” private pay options among the more affluent as they move into public provision (Bassok, Fitzpatrick, Loeb, & Paglayan, 2013). For example, Bassok, Miller, and Galdo (2014) found that the expansion of Florida’s universal voluntary prekindergarten for 4-year-olds led to a reduction in the number of informal family day care providers. Likewise, Cascio and Schanzenbach (2013) found that enrollment in private preschool declined and enrollment in public preschool increased among families with highly educated parents, following the expansion of Georgia’s and Oklahoma’s universal public preschool program. If this is the case, it is possible that we might see gaps in enrollment between high-income families and others (both middle- and low-income families) widening. We might also not see continuing increased enrollment of higher-income children, even though funding continued to expand.

Differences in preschool enrollment rates would be of little interest if preschool were not so strongly linked to children’s subsequent school readiness and early achievement. It is well established that preschool and center-based care improve young children’s early academic skills by providing enriching learning experiences and, sometimes, developmentally appropriate academic instruction. Many early childhood education and preschool programs have been rigorously evaluated. Although estimates differ across particular programs and populations studied, in general these programs increase children’s early academic and cognitive skills. A meta-analysis by Leak and colleagues (2012) of 65 methodologically rigorous studies (20 of which were experimental) conducted between 1967 and 2007 estimated an end-of-treatment effect size of about 0.33 for cognitive and achievement outcomes. They also found that effects decline over time after the treatments ends, with rates of decline of approximately 0.03 per year since end of treatment. Several high-quality studies also find that preschool attendance is related to important reductions in special education placement and grade retention and to improvements in educational attainment (for a review, see Magnuson & Shager, 2010). Recent evaluations of scaled-up state and local pre-kindergarten programs have consistently reported positive effects on children’s school readiness at program completion, with some effects persisting over time (Gormley, Gayer, Phillips, & Dawson, 2005; Gormley, Gayer, & Phillips, 2008; Weiland & Yoshikawa, 2013; Wong, Cook, Barnett, & Jung, 2008) but others fading out during the early school years (Lipsey, Hofer, Dong, Farran, & Bilbrey, 2013).

Low-income children may benefit more than higher-income children from enrollment (Magnuson, Meyers, Ruhm, & Waldfogel, 2004). For example, in Tulsa, Oklahoma, across multiple cohorts of students, researchers found substantial benefits from prekindergarten participation for children from all backgrounds, with slightly larger gains in language, literacy, and math for children from poor versus middle-class families (Gormley et al., 2005; Gormley et al., 2008). In Boston, Massachusetts, researchers found that all children improved their language, literacy, and mathematics outcomes, as well as emotional development and some executive functioning outcomes, as a result of prekindergarten; impacts were significantly larger on some, but not all, assessments for children from low-income families (Weiland & Yoshikawa, 2013).

Although the prior studies by Bainbridge and colleagues (2005) and Magnuson and colleagues (2007) are suggestive that differential preschool enrollment trends coincide with the increasing income-related achievement gaps described by Reardon (2011), they do not cover the period post-2000, and they do not provide fine-grained-enough information to thoroughly scrutinize the role of preschool for low-, middle-, and high-income children. This study extends this previous work, using the same data series but updating it with more recent years and more-fine-grained income categories. Our primary focus is on income-related gaps in enrollment, but to put those results in context, we also provide some results for trends in enrollment by race/ethnicity.

## Data

We use data from the annual October CPS, which has been fielded annually since 1968. The October CPS collects demographic information from parents and includes an education module that tracks school enrollment by asking respondents whether children age ≥3 years attend school. We focus our analyses on children aged 3 and 4 years (since most children enter kindergarten during their 5th year).

### Enrollment in Early Childhood Education

As discussed, preschool-age children in the United States participate in many different types of early childhood education programs as well as informal child care arrangements. The former category includes any form of school or center-based care (including preschools, day care centers, nursery schools, Head Start programs, and state or local prekindergarten programs), whereas the latter includes informal child care in the child’s home or another person’s home (by a baby-sitter, nanny, relative, nonrelative, or family child care provider). The October CPS does not ask parents specifically about participation in early childhood education or informal child care. Instead, it asks (for all children aged ≥3 years) whether the child attends school. It is important therefore to determine what type of arrangements parents have in mind when they report that a 3- or 4-year-old is attending school.

To do so, it is useful to compare results from the October CPS measure of school enrollment with results from measures of early childhood education used in other surveys. In 1998–1999, a total of four such surveys were fielded. Comparisons of the 1999 October CPS data with detailed data from the National Household Education Survey (NHES) for 1999, the Early Childhood Longitudinal Study (ECLS) kindergarten cohort for 1998, and the National Survey of America’s Families (NSAF) for 1999 indicate that the question on school enrollment in the October CPS is capturing information similar to that obtained in other studies (for details, see Magnuson et al., 2007). For example, estimates from the NHES on the proportions of 3- and 4-year-olds attending early childhood education in 1999 (i.e., day care centers, nursery schools, prekindergartens, preschools, and Head Start programs; 46% for 3-year-olds and 70% for 4-year-olds) are quite similar to, though slightly higher than, the October CPS estimates of 3- and 4-year-old children attending school for that year (40% and 68%, respectively). An analysis of the ECLS–K data yields comparable estimates for 4-year-olds’ attendance in early childhood education (69%) in 1997. Findings from the NSAF parallel those from the other sources for 3-year-olds (38% attend early childhood education), although rates of enrollment for 4-year-olds are slightly lower (61%). The NSAF’s lower enrollment rates for 4-year-olds probably reflect a discrepancy in the timing of the assessment: The NSAF was administered throughout the year; thus, some respondents were surveyed during the summer months when educational programs may not have been in session. A comparison of available data sources for a more recent year (2012) suggests that the NHES continues to provide similar, though slightly higher, estimates of preschool enrollment when compared with the October CPS (70% vs. 66% for 4-year-olds and 48% vs. 40% for 3-year-olds).

In sum, the October CPS measure used here is likely to capture nearly all forms of early childhood education. It also seems likely that parents in the October CPS do not identify informal child care and family child care as school, even when such care is licensed. Thus, although the annual October CPS differs from other studies by asking about school rather than fielding a detailed set of questions about early childhood education and nonparental child care arrangements, it yields similar information about 3- and 4-year-old children’s participation in early childhood education. Thus, we rely mainly on the October CPS in this article. However, as a robustness check, we also provide some supplemental results using the NHES Early Childhood Program Participation Survey, which asks respondents specifically about child care and early childhood education but only in selected years (1991, 1995, 1999, 2001, 2005, and 2012).

It is important to note one question change in the annual October CPS. Prior to 1994, parents were asked, “Does your child attend regular school?” In 1994, the CPS added a prompt to clarify that “regular school includes nursery school, kindergarten or elementary school and schooling which leads to a high school diploma.” The addition of this prompt might influence reported enrollment rates and in particular might lead to an increase in reported enrollment in 1994. We present 3-year moving averages in all our analyses to smooth minor fluctuations, but we do not smooth over the 1994 question change when a jump in reported enrollment occurs because of it.

The October CPS also asks respondents whether the school attended was public or private. We use that data to code children as having attended public preschool. This is useful in indicating the public provision of preschool, which we know expanded over this period, although exactly how parents identify their preschool programs as public and private in an era of blended program funding is somewhat unclear.

### Income

The October CPS collects categorical income data by asking respondents which income range represents the total combined income of all members of the family residing in the household during the preceding 12 months. We use the categorical data to classify children into income quintiles for families in households with children aged 3 and 4 years. Quintile 1 includes children from families in the lowest quintile of the family income distribution (i.e., the lowest 20%); Quintile 2, the second-lowest quintile (i.e., the second lowest 20%); and so on, up to Quintile 5, which includes children from families in the highest quintile of the family income distribution (i.e., the highest 20%).

We are particularly interested in how gaps have changed between the bottom and top income families and between the middle and top income families. Accordingly, we use quintiles rather than quartiles (as in Bainbridge et al., 2005) so that we can cleanly identify families at the bottom (Quintile 1), middle (Quintile 3), and top (Quintile 5). We do not use income deciles (as in Reardon, 2011) due to concerns about small sample sizes. In 2013, incomes for families in the top quintile were approximately half between $100,000 and $150,000 and half >$150,000; incomes for the middle quintile ranged from $35,000 to $75,000; and incomes for the bottom quintile ranged from <$5,000 to $25,000 (all amounts in 2013 dollars).

Because of inflation, income categories in the October CPS are not strictly comparable across years. However, if the rank order from rich to poor is roughly correct in each year, low-income families can be reliably distinguished from their middle- or high-income peers. To create quintiles, we must split some categories of income, and we do so essentially by randomly selecting families to be in the higher or lower quintile.

### Race/Ethnicity

Although our primary focus is on income-related gaps, we also carry out some supplemental analyses of gaps related to race/ethnicity. In these analyses, we divide children into four mutually exclusive categories: White non-Hispanic (72% of 3- and 4-year-olds in our sample), Black non-Hispanic (13%), Hispanic (10%), or other (5%). Because the last category represents very small numbers of children, we focus on the first three groups when analyzing enrollment by race/ethnicity. We note also that our analyses of Hispanic children begin in 1973 because they are not identified separately in the October CPS before that year.

## Results

**FIGURE 1.**
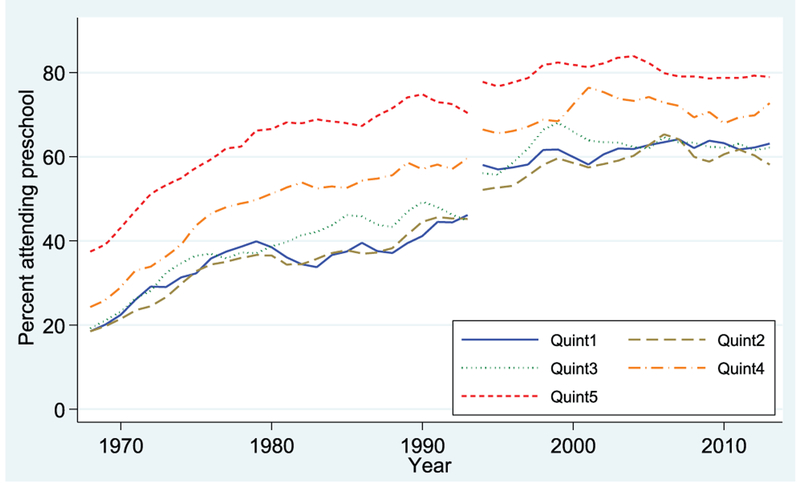
Percentage of children enrolled in preschool, by family income quintile: 4-year-olds. Data from October Current Population Survey (3-year moving averages).

We begin by considering the trends over time in the share of 4- and 3-year-old children enrolled in early childhood education (which we refer to as *preschool* for brevity) by income quintile. Figure 1, for 4-year-olds, shows an upward trend in enrollment over time for all five income groups, but the enrollment of children from the top two income groups (Q4 and Q5) is higher than that of the other three groups throughout the period. By 2013, the last year of our time series, nearly 80% of 4-year-olds from the highest income quintile (Q5) are enrolled in preschool, compared with roughly 60% of their peers from middle- (Q3) and low-income (Q1) families.

**FIGURE 2.**
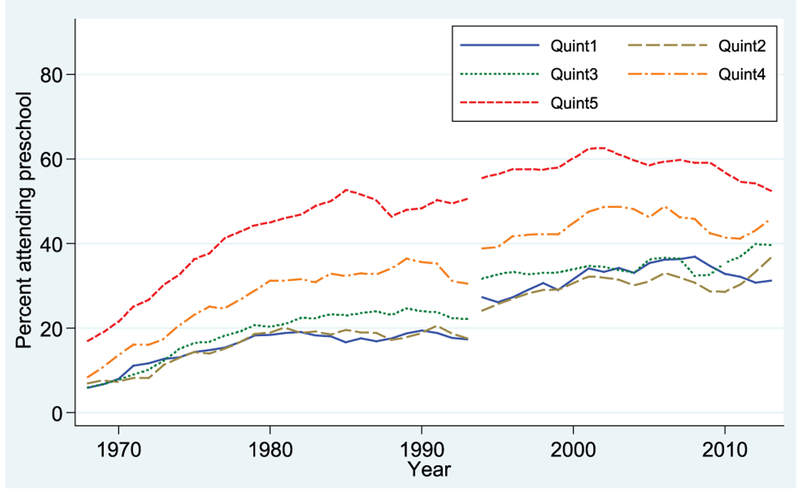
Percentage of children enrolled in preschool, by family income quintile: 3-year-olds. Data from October Current Population Survey (3-year moving averages).

Levels of enrollment are lower for 3-year-olds (Figure 2) throughout the period. But for this age group as well, enrollment has risen considerably since 1968. By 2013, more than half of 3-year-olds from Q5 are enrolled in preschool, as compared with fewer than one-fifth in 1968, while enrollment of the lowest-income children (Q1) rose from about 5% to about 30% over the period. However, it is difficult to assess the magnitude of the gaps among income groups and the changes in the gaps by looking at overall trends. For that reason, we next turn to a series of figures that graph the gap in the percentage enrolled between two specific income groups. To do so, we ran a series of regression models in which we predicted a child’s enrollment in early childhood education as a function of the family’s income quintile group. For example, to estimate the gap in enrollment between children in the lowest-income group (Q1) and the highest (Q5), we ran a regression model for children from those two income groups, including a dummy variable for Q5 (so that Q1 was the reference category), with data pooled for three consecutive years. The coefficient on Q5 indicates the difference in the enrollment rate between children in Q5 and children in Q1 for that 3-year period. Similar regression models were used to produce estimates comparing enrollment in the middle-income group (Q3) and the highest (Q5).

**FIGURE 3.**
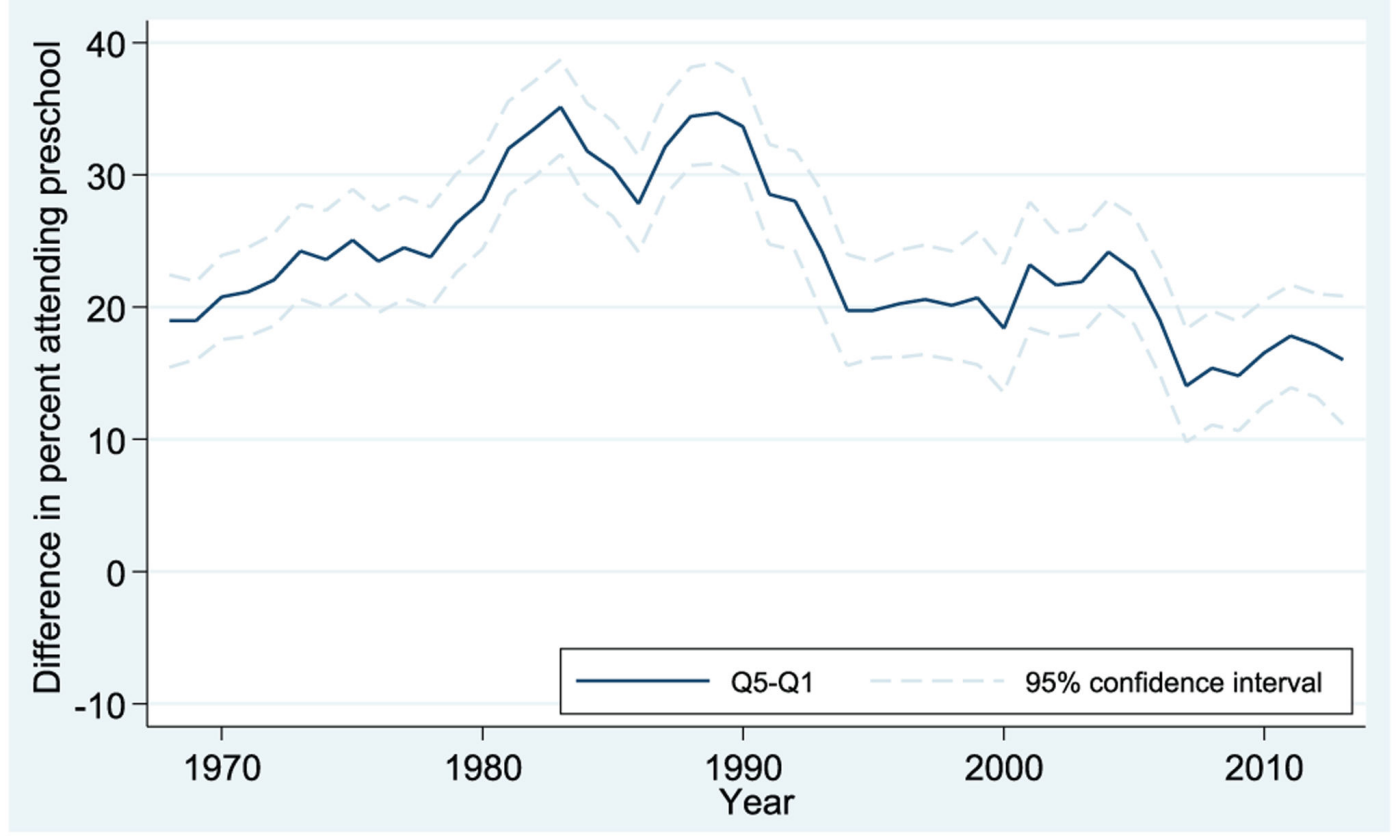
Preschool enrollment gaps between first and fifth income quintiles: Q5 vs. Q1, 4-year-olds. Data from October Current Population Survey (3-year moving averages).

**FIGURE 4.**
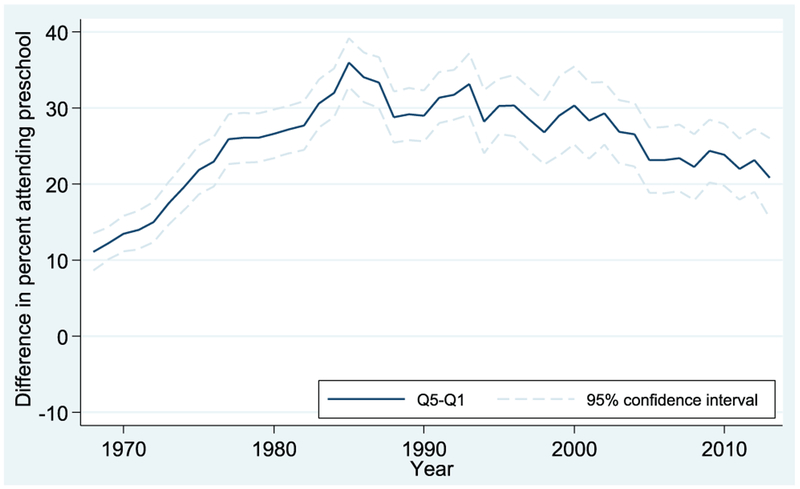
Preschool enrollment gaps between first and fifth income quintiles: Q5 vs. Q1, 3-year-olds. Data from October Current Population Survey (3-year moving averages).

Results for the Q5-Q1 comparison (i.e., comparing the highest-income children to the lowest-income children) are shown in Figures 3 and 4 for 4-year-olds and 3-year-olds, respectively. The dark lines represent the enrollment gap of Q5 relative to Q1, and the shaded lines indicate the 95% confidence intervals for those estimates.

In Figure 3, for 4-year-olds, a steady increase in inequality is apparent in the 1970s and 1980s, as the gap rises from about 20 percentage points to >30 percentage points. This trend appears to slow or even reverse starting in the late 1980s and early 1990s, with further convergence apparent from about 2005 onward. By the end of the time series, in 2013, the gap is about 15 percentage points.

The story is different for 3-year-olds, as shown in Figure 4. The Q5-Q1 gap rises from its starting level of 10 percentage points in 1968 to a high of >30 percentage points in the mid-1980s. The gap then declines a bit in the succeeding decades but remains elevated relative to its 1968 level, at just >20 percentage points in 2013.

**FIGURE 5.**
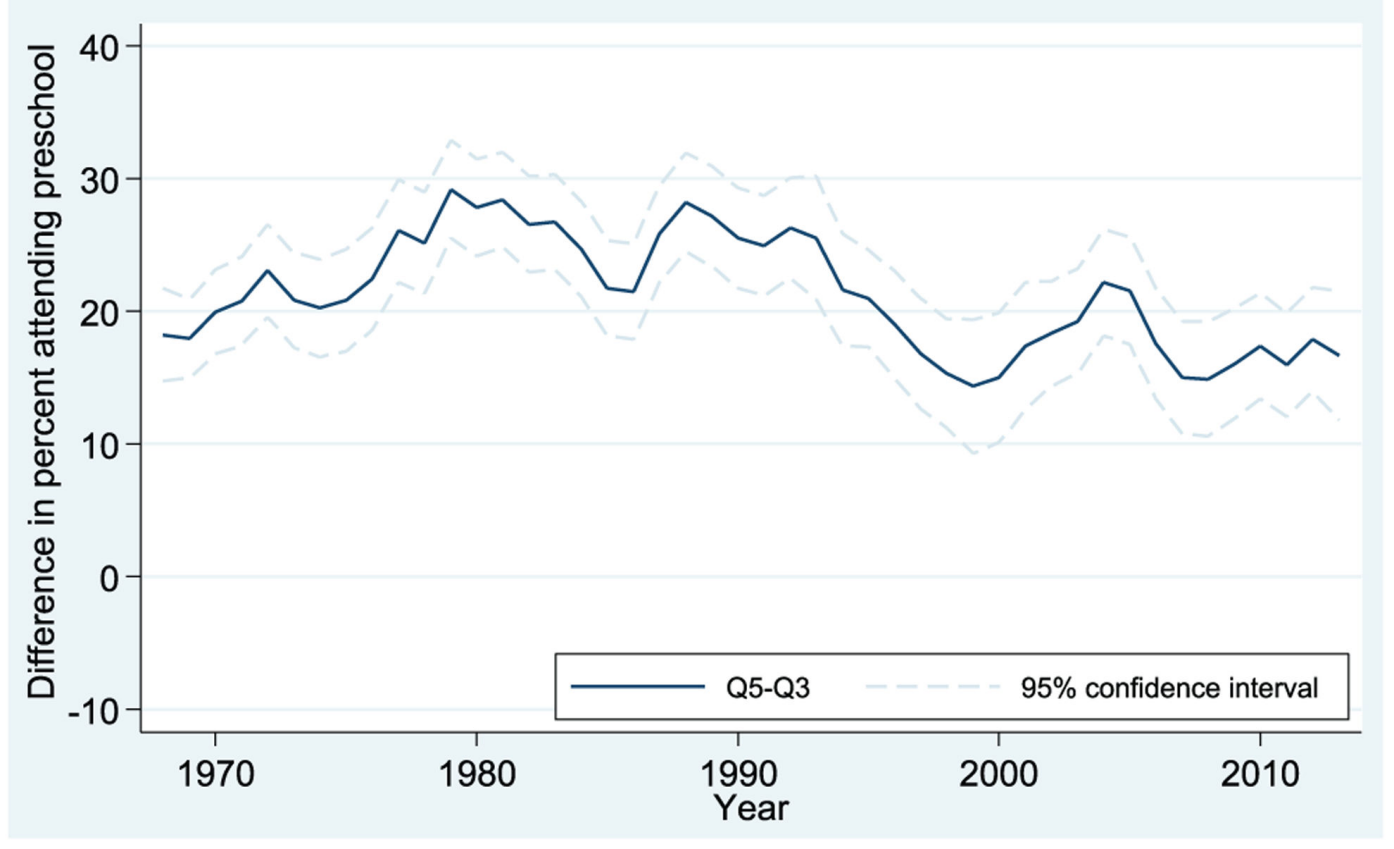
Preschool enrollment gaps between third and fifth income quintiles: Q5 vs. Q3, 4-year-olds. Data from October Current Population Survey (3-year moving averages).

**FIGURE 6.**
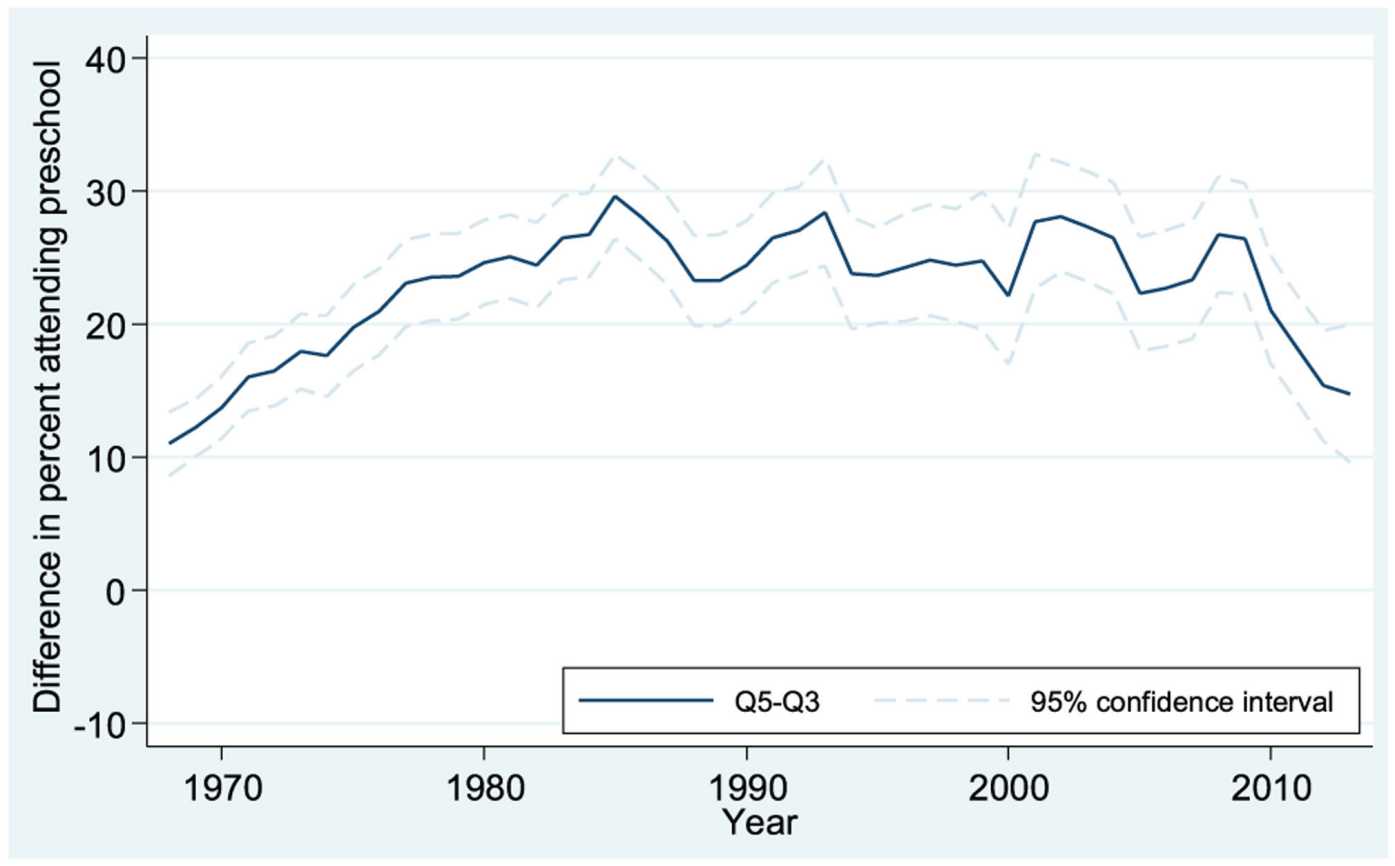
Preschool enrollment gaps between third and fifth income quintiles: Q5 vs. Q3, 3-year-olds. Data from October Current Population Survey (3-year moving averages).

Estimates for Q5-Q3 gaps (comparing the highest-income children to the middle-income group) are shown in Figures 5 and 6 for 4- and 3-year-olds, respectively. Looking first at Figure 5, for 4-year-olds, we can see that the Q5-Q3 gap follows a somewhat similar, though more muted, pattern to that seen for the Q5-Q1 gap in Figure 3. The high- to middle-income enrollment gap widens from about 20 percentage points in 1968 to a high of around 30 percentage points in the early 1980s and then declines to a gap of around 15 percentage points in 2013.

Looking at Figure 6, for 3-year-olds, we see here as well that trends in the gaps between high- and middle-income children mirror those for high- versus low-income children but again are more muted. The Q5-Q3 gap starts at around 10 percentage points in 1968, rises to a high of nearly 30 percentage points in the mid-1980s, and then declines to roughly 15 percentage points at the end of the series in 2013.

**APPENDIX FIGURE 1.**
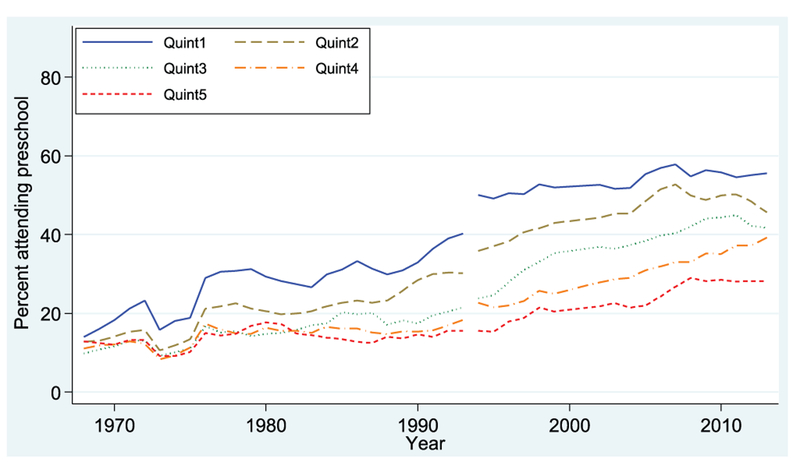
Percentage of children enrolled in public preschool, by family income quintile: 4-year-olds. Data from October Current Population Survey (3-year moving averages).

**APPENDIX FIGURE 2.**
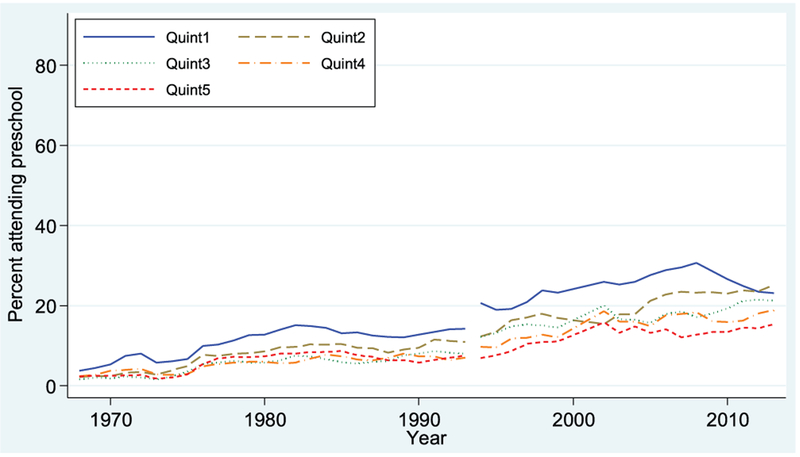
Percentage of children enrolled in public preschool, by family income quintile: 3-year-olds. Data from October Current Population Survey (3-year moving averages).

Taking advantage of the data on public and private enrollment, we examined whether the gaps just discussed may have been explained by differences in provision of public funding. We find that as expected, among children enrolled in preschool, 4- and 3-year children in the lowest income quartile have the highest rates of enrollment in public programs (Appendix Figures 1 and 2), with most of their overall enrollment consisting of public programs. Among the lowest income quintiles, the share of children in public programs grew the most through the 1970s and early 1980s and has been largely flat since the mid-1990s, with at most very modest increases. In contrast, the higher-income quintiles, especially Quintiles 4 and 5, demonstrated consistently low enrollment through the 1990s and only in the most recent years have seen modest increases in public enrollment.

### Supplemental Results on Income-Related Gaps in the NHES

**APPENDIX FIGURE 3.**
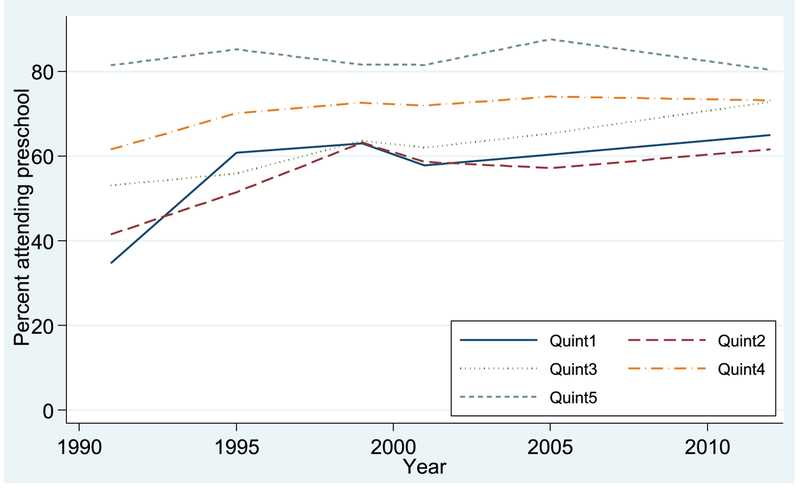
Percentage of children enrolled in preschool, by family income quintile: 4-year-olds. Data from National Household Education Survey.

**APPENDIX FIGURE 4.**
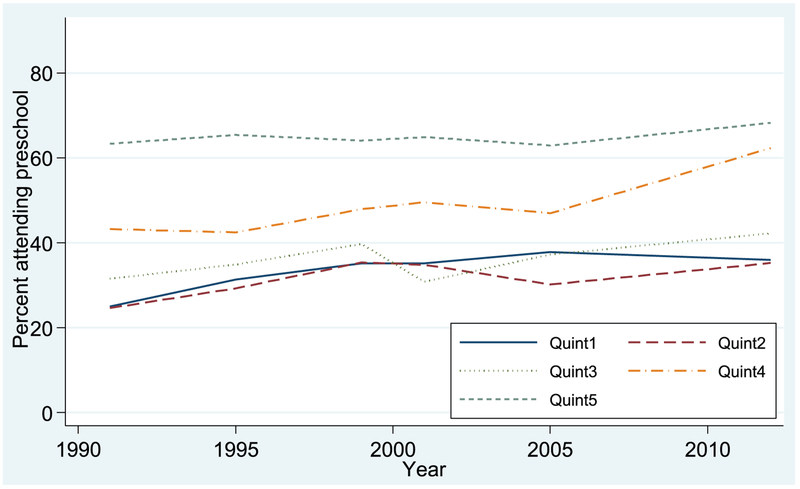
Percentage of children enrolled in preschool, by family income quintile: 3-year-olds. Data from National Household Education Survey.

**APPENDIX FIGURE 5.**
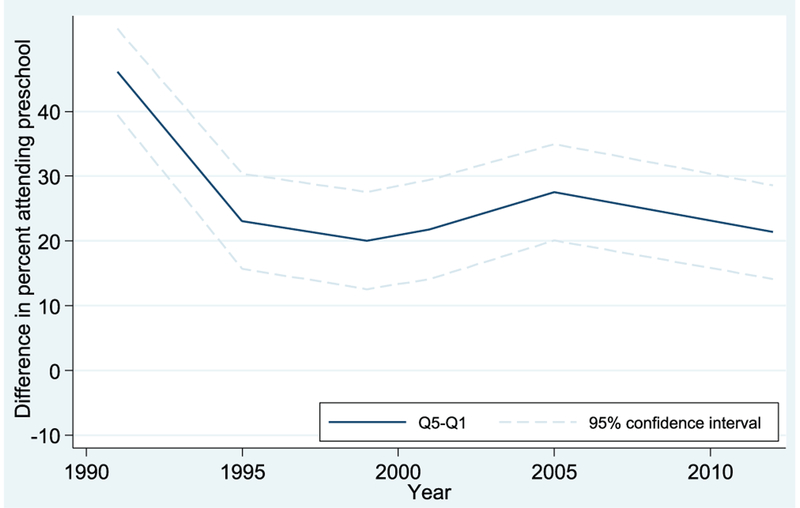
Preschool enrollment gaps between first and fifth income quintiles: Q5 vs. Q1, 4-year-olds. Data from National Household Education Survey.

**APPENDIX FIGURE 6.**
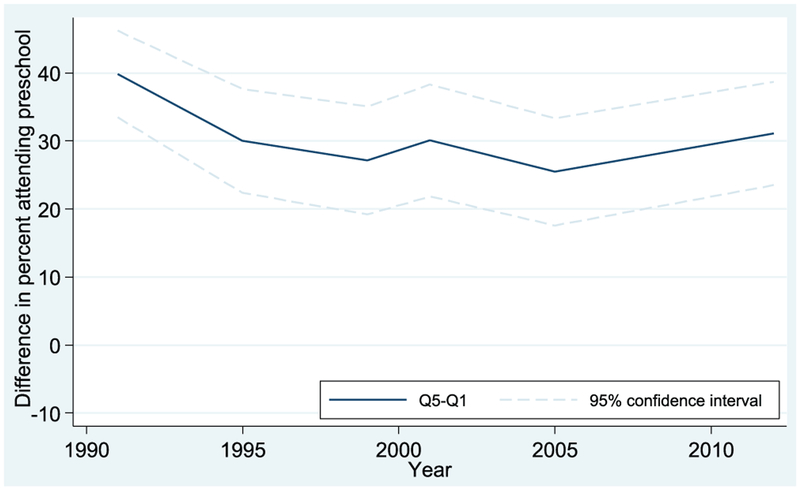
Preschool enrollment gaps between first and fifth income quintiles: Q5 vs. Q1, 3-year-olds. Data from National Household Education Survey.

As a robustness check, we examined trends in income-related gaps in enrollment in the NHES Early Childhood Program Participation Survey, which asked respondents about child care and early childhood education in 1991, 1995, 1999, 2001, 2005, and 2012. These results, shown in Appendix Figures 3–6, show slightly larger gaps in the early 1990s than those in the CPS but portray trends similar to those found in the October CPS for those years. For example, as in the October CPS, the NHES data show the Q5-Q1 gaps for 3- and 4-year-olds declining in the 1990s but leveling off thereafter.

### Supplemental Results on Gaps by Race/Ethnicity

**APPENDIX FIGURE 7.**
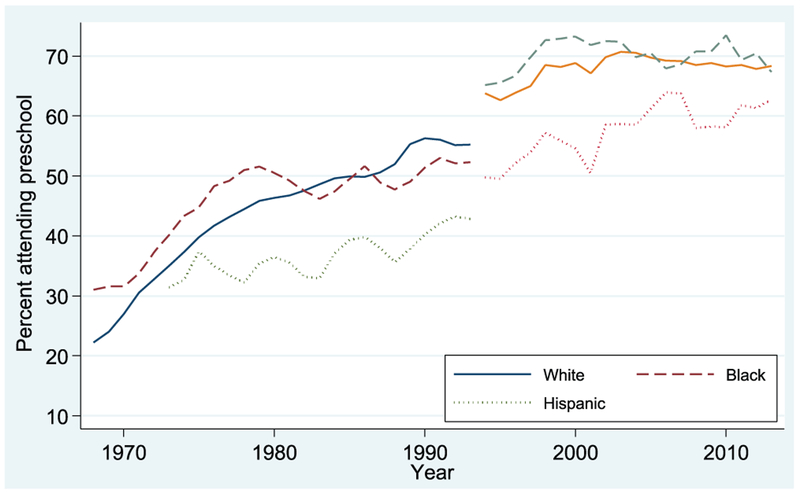
Percentage of children enrolled in preschool, by race: 4-year-olds. Data from October Current Population Survey (3-year moving averages).

**APPENDIX FIGURE 8.**
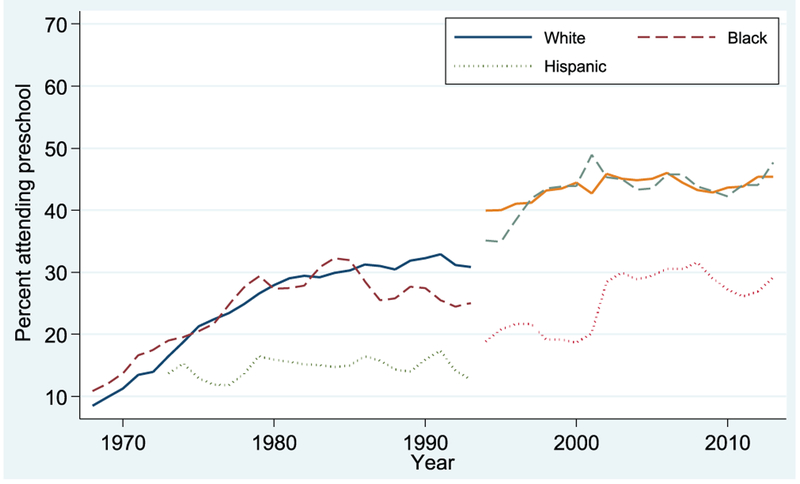
Percentage of children enrolled in preschool, by race: 3-year-olds. Data from October Current Population Survey (3-year moving averages).

**APPENDIX FIGURE 9.**
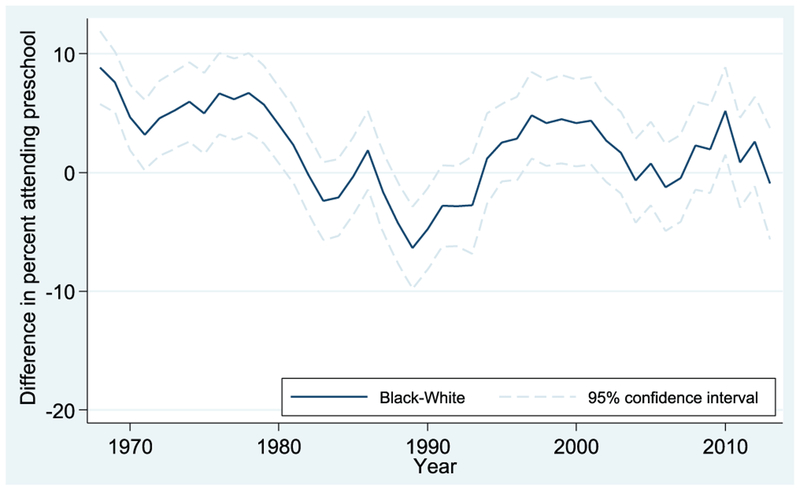
Preschool enrollment gap between White and Black children: 4-year-olds. Data from October Current Population Survey (3-year moving averages).

**APPENDIX FIGURE 10.**
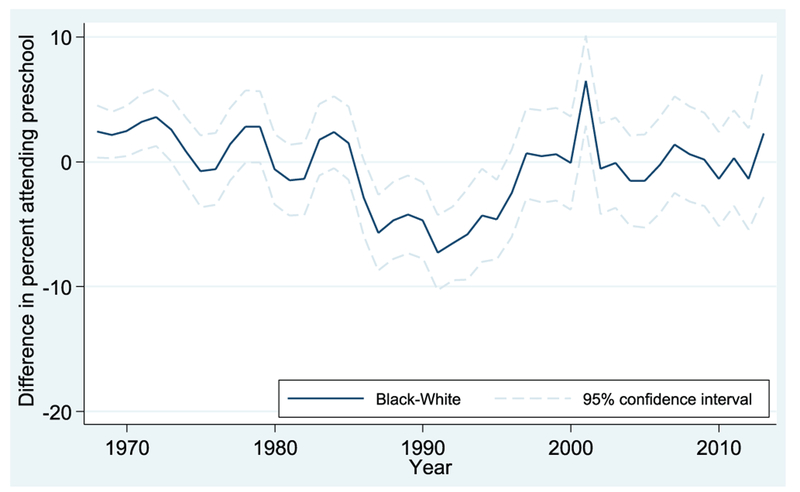
Preschool enrollment gap between White and Black children: 3-year-olds. Data from October Current Population Survey (3-year moving averages).

**APPENDIX FIGURE 11.**
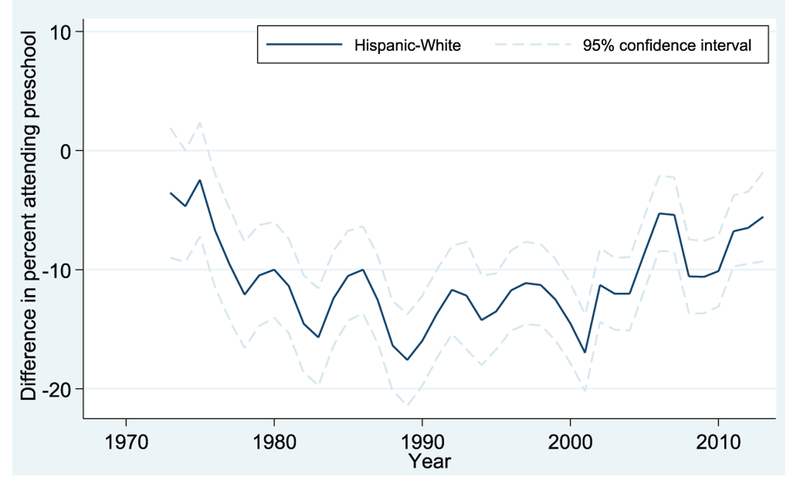
Preschool enrollment gap between White and Hispanic children: 4-year-olds. Data from October Current Population Survey (3-year moving averages).

**APPENDIX FIGURE 12.**
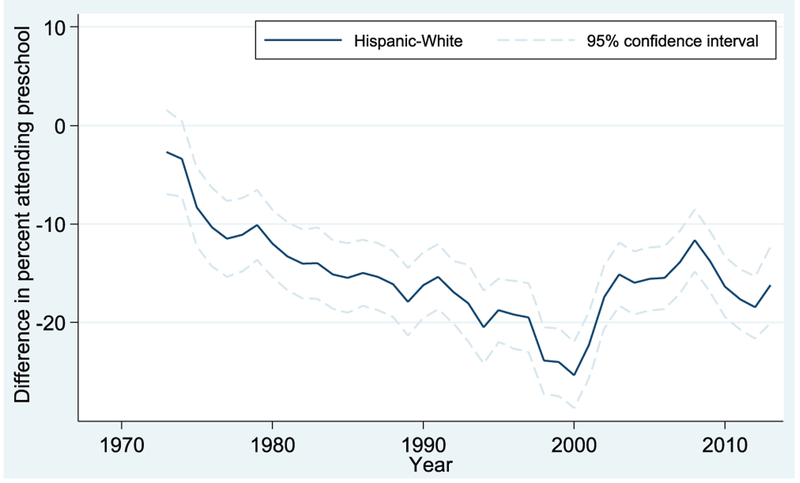
Preschool enrollment gap between White and Hispanic children: 3-year-olds. Data from October Current Population Survey (3-year moving averages).

In the appendix, we also include a set of figures displaying trends in enrollment for White, Black, and Hispanic children, as well as trends in the Black-White gaps and Hispanic-White gaps (Appendix Figures 7–12). These graphs show that White 3- and 4-year-olds are less likely to be enrolled than their Black counterparts until the mid-1980s, at which point their rate of enrollment exceeds that of Black children (because Black children’s enrollment plateaus). Both groups see a jump in enrollment in the early 1990s (perhaps associated with the change in the enrollment question in 1994), after which Black children’s enrollment rates again are higher. Patterns when the Black-White gaps in enrollment are graphed are similar to those in the Q5-Q1 graphs, perhaps not surprising since Black children are disproportionately represented in Q1 (in our data, about 30% of Q1 is Black), while White children make up the majority of children in Q5 (in our data, 86% of Q5 is White).

Patterns for Hispanic children and Hispanic-White gaps are very different. Enrollment for Hispanic children is low relative to that of both White and Black children throughout the period. The gap in the enrollment of 3-year-old Hispanic children relative to White children grows until about 2000, at which point it reverses and begins to narrow. The same pattern is evident for 4-year-olds but with considerably more variability in the data.

## Discussion

These results shed some light on the main question that we raised at the outset—the extent to which the trends in income-related inequality in enrollment in early childhood education are consistent with what we know about trends in inequality of academic achievement. In particular, do these results for income-related gaps in preschool enrollment between the highest- and lowest-income children line up with what we know about subsequent gaps in school achievement for relevant cohorts?

To summarize the enrollment patterns, we find growing income-related gaps in enrollment in early education between 3- and 4-year-old children from Q1 (the lowest income quintile) and Q5 (the highest income quintile) in the 1970s and 1980s, followed by stable (for 3-year-olds) or declining (for 4-year-olds) gaps thereafter. If these gaps are predictive of subsequent inequality in school achievement, we should see growing inequality of achievement between low- and high-income children born in the 1970s and 1980s, followed by smaller gaps for cohorts born in the 1990s or later.

The most direct evidence on this point comes from Reardon’s (2011) Figures 5.1 and 5.2, showing trends in the 90/10 income achievement gaps in reading and math, respectively, by birth cohort. Reardon’s data point to growing reading and math gaps for children born in the 1970s and 1980s, which is consistent with the patterns that we see with regard to early education enrollment for those cohorts. However, Reardon’s data show inequality continuing to rise for the ECLS kindergarten cohort born in the early 1990s, then leveling off or falling for the ECLS birth cohort born in the early years from 2000 onward. Not available at the time for Reardon’s analysis, there is additional evidence now from the most recent ECLS kindergarten cohort of 2010 showing that income gaps in achievement appear to be stable or slightly declining from 1998 through 2010 (Bassok & Latham, 2014; Magnuson & Duncan, 2014; Reardon & Portilla, 2015).

Thus, our data on enrollment for Q1 and Q5 seem to line up fairly well with Reardon’s data on achievement in the early decades and the most recent years, but the increasing gaps during the 1990s are not aligned well with the increasing preschool enrollment (and declining income-related gaps in enrollment).

However, the expected magnitude of changes in pre-school enrollment would likely have led to a rather small convergence of test scores. The Q1-Q5 enrollment gap peaked at >30 percentage points during the middle of the 1980s and then declined to roughly 15 percentage points after 2010. The magnitude of the expected convergence of test scores would likely be modest, however, because it would reflect both the reduction in the enrollment gap and the overall program effects. For example, if the effect of the programs were on average about 0.33 standard deviation, then a 15–percentage point decline in the gap would yield approximately a .05 improvement in low-income children’s achievement (.15 × .33 = .0495). In addition, we would not expect perfect congruence with achievement test trends, given that many factors other than early childhood education enrollment influence school achievement and that there are aspects of preschool, such as quality and hours in care, that we do not observe in our data. Indeed, it would be surprising if trends in early childhood education enrollment alone fully predicted trends in academic achievement.

Our results also shed some light on how enrollment gaps between middle- and high-income children may have changed over this period. Overall patterns for the Q3-Q5 gaps are similar to those seen for the Q1-Q5 gaps, although the growth in the gaps in the 1980s does tend to be more muted when comparing Q3 to Q5 than Q1 to Q5. Again, these results line up moderately well with Reardon’s data (2011); however, his Q3-Q5 gaps in reading and math achievement rise steadily throughout this period (see his Figures 5.7 and 5.8), whereas we find inequality in enrollment either stable or falling in the most recent decades. However, in more recent data, Reardon and Portilla (2015) find the 90/50 gaps in achievement lower in 2010 than in 1998 or 2006–2007, which would be more consistent with the enrollment patterns that we find.

What factors might explain the changes in inequality of enrollment in early childhood education that we observe over the 1968–2013 period and, in particular, the dramatic changes in the gap in enrollment between the highest- and lowest-income children? Although a full analysis of the causes of the trends in this enrollment gap is beyond the scope of this article, it is informative to briefly consider what we know about trends in high-income families’ spending on early childhood education and trends over time in public policies affecting enrollment of children from low-income families.

It is well established that high-income families spend more in absolute terms on items for children than do lower-income families, because they have more resources to invest in their children and also because they may have different preferences on how to spend the resources they do have (Becker, 1991; Chin & Phillips, 2004; Kalil & DeLeire, 2004; Lareau, 2003). Analyses of Consumer Expenditure Survey data for 1997–2006 find that families in the highest income quintile spent >$1,200/year on average on child care while families in the lowest income quintile spent on average <$100/year (Kaushal, Magnuson, & Waldfogel, 2011). We do not know the extent to which this gap in expenditures on child care has grown over time. However, we do know that the income-related gap in overall expenditures on child enrichment items has grown substantially over time, as overall income inequality has grown. Analyses of Consumer Expenditure Survey data show average annual spending on child investment items growing from $3,536 to $8,872 between the early 1970s and the early years from 2000 onward for the highest–income quintile families while growing from only $835 to $1,315 for the lowest–income quintile families (Duncan & Murnane, 2011).

Changes in enrollment of children from the lowest-income families are likely influenced not just by changes in their families’ incomes and expenditure patterns but also by changes over time in the availability of public funding through programs such as child care subsidies, tax credits, and direct support for programs such as Head Start and public prekindergarten. Child care subsidy spending was modest until the welfare reforms of the late 1980s and 1990s, at which point it accelerated and overtook spending on the longer-established child care tax credit programs (Meyers, Rosenbaum, Ruhm, & Waldfogel, 2004). Head Start spending and associated enrollment have also exhibited considerable variability over time. Data from the federal Office of Head Start (2014) indicate that from its base of nearly 500,000 children enrolled in 1970, enrollment in Head Start fell throughout the 1970s (except for a onetime increase in 1979) and then stagnated at around 400,000 in the early 1980s before growing modestly in the late 1980s to a level of about 450,000 in 1989. From 1990 onward, enrollment rose steadily to approximately 900,000 children in 2001, a level that it remained at throughout the years from 2000 onward. State-funded public prekindergarten programs are a relatively newer form of subsidy for early education and expanded rapidly in the past decade, as the share of 4-year-olds enrolled in prekindergarten doubled from 14% in 2002 to 29% in 2014 (National Institute for Early Education Research, 2014).

In the absence of a comprehensive analysis that takes into account changes in all forms of public funding for child care and preschool from 1968 to 2013, we do not know what role changes in public spending may have had in explaining the trends in enrollment of low-income children that we have examined in this paper. Analyses of specific periods suggest that the influence of public policies can be substantial. For instance, a study of data from 1992 to 2000 finds that child care subsidies per low-income child tripled while Head Start spending per poor child doubled and that these funding increases explained 8 to 11 percentage points of the 16–percentage point increase in early childhood education enrollment for low-income 3- and 4-year-olds over this period (Magnuson et al., 2007). Yet, analysis of state spending in Florida, Georgia, and Oklahoma points to public funding at least partially crowding out private funding (Bassok et al., 2014; Cascio & Schanzenbach, 2013).

Our analysis finds that publicly funded programs have been the predominant auspice of enrollment for most families with incomes in the lowest quintile since the 1980s. Of children enrolled in preschool, however, the percentage in public programs has largely remained constant since the mid-1990s. Also noteworthy, however, is the higher number of children in the highest income quintiles that attend public programs, as well as how low-income 3-year-olds are much less likely to be in public programs than 4-year-olds (among those who are attending preschool).

## Conclusion

Is the enrollment of children in early childhood education more or less strongly linked to family income now than it was in the past? The answer depends on the income and age groups examined and also the period.

When we compare children from the most affluent families with those from the lowest income group (the Q5-Q1 comparison), the gap in enrollment for 3-year-olds is larger now than it was 45 years ago—although not as large as it was at its peak in the 1980s. The comparison for 4-year-olds is perhaps more encouraging in that the gap now is not larger than it was 40 years ago and smaller than it was in the 1980s. We also see sizable gaps between high-income children and the middle-income group, again with some evidence of the groups pulling apart over time for 3-year-olds, although not for 4-year-olds.

Such sizable and persistent gaps in an investment believed to affect school readiness should be of concern. But it is not yet clear how consequential these gaps are for subsequent gaps in school achievement (or gaps in other outcomes). Our analysis points to different trends for 3- and 4-year-olds, so the implications of these trends will depend on the relative importance of enrollment at these two ages. Moreover, our analysis is limited in that we do not have data about changes in the quality of provision or the effects of early education on later achievement.

If growing inequality in early childhood education enrollment is contributing to widening inequality of school achievement, there may be a role for policy remedies. But at this point, it is not clear what the appropriate policy response should be. There is not a consensus in the field about the relative merits of universal versus targeted early childhood education policies. Esping-Andersen (2004) and others (e.g., Bradbury et al., 2015) argue that the most appropriate response is for government to increase its investment in universal preschool programs that allow children of all income levels to attend educationally oriented preschool in the year or two before school entry. This approach is predicated on the idea that if child care is left to the market, inequalities will emerge, and that if preschool is universally provided, low-income children will benefit more from that provision than will more advantaged children. In addition, the participation of more affluent children in a universal program is often thought to build broader political support for the program. But others advocate for increasing child care subsidies aimed at increasing the affordability of early education for low-income families and/or expanding specific programs targeted to low-income families such as Head Start (Duncan & Magnuson, 2003). The rationale for a more targeted approach is that it would ensure that low-income children, not affluent children, benefit from the program expansions.

The recent expansion of public prekindergarten programs can perhaps be seen as a compromise between these two policy positions. Often such programs, while universal, are phased in, beginning with the most disadvantaged communities and schools to ensure that when funds are limited, the most disadvantaged children are served first. As we have seen, these expansions, focused on 4-year-olds, have increased the share of children enrolled in recent decades and have likely helped reduce gaps in enrollment between low- and high-income children. But it is also clear from the data presented that sizable disparities in early enrollment remain.
